# Exploring Brain Dynamics via EEG and Steady-State Activation Map Networks in Music Composition

**DOI:** 10.3390/brainsci14030216

**Published:** 2024-02-26

**Authors:** Xiaohu Gu, Leqi Jiang, Hao Chen, Ming Li, Chang Liu

**Affiliations:** 1 Laboratory of Image Processing and Pattern Recognition, School of Information Engineering, Nanchang Hangkong University, Nanchang 330038, China; 2104081000008@stu.nchu.edu.cn (X.G.); jdz_jlq@nchu.edu.cn (L.J.); liming@nchu.edu.cn (M.L.); lcsszz@nchu.edu.cn (C.L.); 2 School of Information Engineering, Nanchang Hangkong University, Nanchang 330038, China

**Keywords:** steady-state activation map (SSAM), music creation, EEG state recognition

## Abstract

In recent years, the integration of brain–computer interface technology and neural networks in the field of music generation has garnered widespread attention. These studies aimed to extract individual-specific emotional and state information from electroencephalogram (EEG) signals to generate unique musical compositions. While existing research has focused primarily on brain regions associated with emotions, this study extends this research to brain regions related to musical composition. To this end, a novel neural network model incorporating attention mechanisms and steady-state activation mapping (SSAM) was proposed. In this model, the self-attention module enhances task-related information in the current state matrix, while the extended attention module captures the importance of state matrices over different time frames. Additionally, a convolutional neural network layer is used to capture spatial information. Finally, the ECA module integrates the frequency information learned by the model in each of the four frequency bands, mapping these by learning their complementary frequency information into the final attention representation. Evaluations conducted on a dataset specifically constructed for this study revealed that the model surpassed representative models in the emotion recognition field, with recognition rate improvements of 1.47% and 3.83% for two different music states. Analysis of the attention matrix indicates that the left frontal lobe and occipital lobe are the most critical brain regions in distinguishing between ‘recall and creation’ states, while FP1, FPZ, O1, OZ, and O2 are the electrodes most related to this state. In our study of the correlations and significances between these areas and other electrodes, we found that individuals with musical training exhibit more extensive functional connectivity across multiple brain regions. This discovery not only deepens our understanding of how musical training can enhance the brain’s ability to work in coordination but also provides crucial guidance for the advancement of brain–computer music generation technologies, particularly in the selection of key brain areas and electrode configurations. We hope our research can guide the work of EEG-based music generation to create better and more personalized music.

## 1. Introduction

### 1.1. Introduction to the Research Areas

Music, an auditory art and intellectual brain activity, conveys emotions and facilitates thought communication, representing advanced cognitive activity [1,2]. Advances in information science and artificial intelligence are transforming music composition, moving from reliance on composers to using technologies like computers and algorithms. This shift not only broadens the technical avenues for music creation but also provides unprecedented opportunities for the public to engage in music composition, thereby opening up new possibilities for research in musicology, cognitive science, and artificial intelligence.

Electroencephalography (EEG), a noninvasive technique sensitive to the brain’s millisecond-level changes [3], combined with BCI technology, captures the brain’s electrical activity with high temporal resolution [4]. Neuroscience studies suggest a significant overlap in the neural mechanisms and patterns activated by musical activities (such as playing or listening to music) and those engaged during the processing of non-musical brain information, such as memory, indicating a shared neural basis between music and general brain information processing [5]. The therapeutic and joyous effect that music has on individuals can be linked to the fact that both music and human physiology are governed by similar natural laws [6]. The technique of mapping brain activity to music by utilizing EEG signals has been demonstrated to be an effective approach [7].

Researchers employ various mapping strategies to convert EEG signals into music of different formats, streamlining the music creation process [8]. Miranda [9] was the first to attempt mapping EEG signals to notes ranging from A4 to A6# and selected appropriate notes based on the power of the electrodes at different times to achieve music generation. Subsequent studies [10] classified the frequency bands of the signals to produce music of different styles. Thomas Deuel [11] and colleagues innovated with the ‘encephalophone’, enabling patients with movement disorders to create music from their brainwaves during rehabilitation, highlighting the profound music–brain connection.

Much of the current research centers on the relationship between brainwaves and music, specifically mapping brainwave features to various musical elements and then integrating these features with musical theory for composition [12]. However, several issues persist: (1) Most datasets merely use music as a stimulus and then employ the resulting music-induced EEG for composition. The similarity between EEGs produced from passive music listening and those produced during active music creation remains an open question. (2) There is also a notable lack of studies examining which specific EEG regions are activated or engaged by musical elements such as pitch and melody during the process of music creation [13].

In light of the ambiguity in mapping brainwave responses to musical elements and the uncertainty regarding the appropriateness of using EEG data from passive listening for active music composition, this study contributes to a clearer understanding of how brainwave responses to musical elements can be interpreted and utilized in this context. To verify the precision of this simulation and to identify the brain regions and electrodes most closely associated with music recall and creation, our work has made the following technical contributions:

- In this study, a self-attention neural network was developed based on steady-state activation mapping (SSAM). Utilizing SSAM, the model effectively identifies and highlights brain region data closely related to and active in current cognitive activities, spanning the entire time frame. Subsequently, the model successfully located these key brain regions and analyzed the complementarity between different frequency bands.
- Addressing the overlooked aspects in the current research, namely the differences in brain activity between external melody stimulation and internal melody recall and creation, our team established a control group comprising individuals with and without musical training. In this paper, a paradigm was independently designed, and EEGs were recorded during both the phase of receiving music melody stimulation and the phase of recalling and creating melodies.
- Experimental results conducted on the aforementioned test set demonstrate that, compared to advanced models in the field of emotion recognition, the proposed model achieved performance improvements of at least 2.94% and 7.65% in untrained general subjects and subjects with a musical background, respectively. Notably, in the recognition of states during music recall and creation, the model exhibited a significant improvement of at least 8%.
- The proposed model successfully identified brain regions closely associated with musical reminiscence and composition and revealed variations in activity across different frequency bands among these regions in different subjects. Furthermore, this paper provides a detailed analysis of the brain region activation patterns and the modes of collaboration among these areas during states of musical reminiscence and composition.

The remainder of this paper is structured as follows: Section 2 consolidates the relevant literature on EEG feature extraction and EEG-to-music conversion and discusses the application of attention mechanisms in EEG-related recognition tasks. Section 3 provides a detailed description of the proposed SSAM network structure and methodology. Section 4 presents the experimental evaluation results, and Section 5 concludes the paper.

### 1.2. Related Work and Motivation

In recent years, the research field of converting EEG signals into music has regained the attention of scholars both domestically and internationally, with researchers striving to establish the mapping relationship between music and EEG signals. Wu et al. [14] undertook systematic research on event-related potentials and introduced a novel brainwave music generation algorithm that was shown to be effective at alleviating orthodontic pain. Further research [15] developed a scale-free brainwave music generation strategy by mapping EEG signal voltage amplitude and power to musical pitch and loudness, leading to the successful creation of various musical styles, including pentatonic scales, heptatonic scales, and quartets. Zerafa [16] utilized steady-state visual-evoked potentials for music generation, creating music from brain signals elicited by visual stimuli.

Advances in machine learning and deep learning have further propelled EEG-based music creation, with researchers developing various models for higher-quality music production and real-time composition, including transforming emotional valence and arousal into music [17] and using P300 ERP for instant note synthesis [18].

The introduction of the attention mechanism aims to address the challenges faced by deep learning models when processing sequence data, particularly long sequences. In recent years, the attention mechanism has been widely applied in EEG classification research. For instance, Chen et al. [19] integrated a hierarchical bidirectional gated recurrent unit (GRU) network with an attention mechanism to derive a more discriminative EEG feature representation. Zhang et al. [20] introduced a convolutional recurrent attention model, aiming to learn advanced EEG representations and further explore temporal dynamics. Qiu et al. [21] developed a specialized attention network tailored to extract complementary features from both EEG and eye movement signals, subsequently applying this network for emotion classification. Li et al. [22] considered the importance of EEG signals from different frequency bands in emotion classification and employed concatenated features across these bands, achieving superior classification results on the DEAP dataset. Giuseppe Varone al. [23] introduced an unsupervised EEG preprocessing pipeline for SMR-based BCIs, which employs pre-trained convolutional neural networks (CNNs) to enhance classification accuracy and reliability in real-time scenarios. Collectively, these studies demonstrated the significant advantages of utilizing attention mechanisms in extracting unique state information from EEGs.

This study is dedicated to exploring domains previously neglected in EEG music composition research. The main objective is to compare the differences in brain region activation between passive music listening and active music recall and creation, identifying the brain regions and electrodes most closely associated with states of musical memory and creation. To achieve these objectives, a new research methodology is developed, in addition to a dataset specifically designed to capture states of music recall and creativity. Additionally, an attention network model based on SSAM is introduced that is capable of analyzing key features of brain regions during the music composition process and precisely identifying the brain areas and electrodes related to the state of music creation. Furthermore, our research investigates the collaborative patterns of brain regions throughout the music creation process, thereby enriching the understanding of the neural mechanisms behind musical creativity.

## 2. Materials and Methods

### 2.1. Materials

#### 2.1.1. Experimental Subjects

We recruited 24 volunteers, aged between 20 and 24 years, with an average age of 22.8 ± 1.6 years. Among the participants, 12 were regular students with no musical training, while the other 12 were students from the conservatory specializing in performance and vocal studies. All participants had normal hearing and vision, were in good health, maintained stable mental states, and had no significant medical or psychiatric history. All participants signed an informed consent form. The specific information is shown in Table 1.

#### 2.1.2. Experimental Equipment

**Figure 1:**
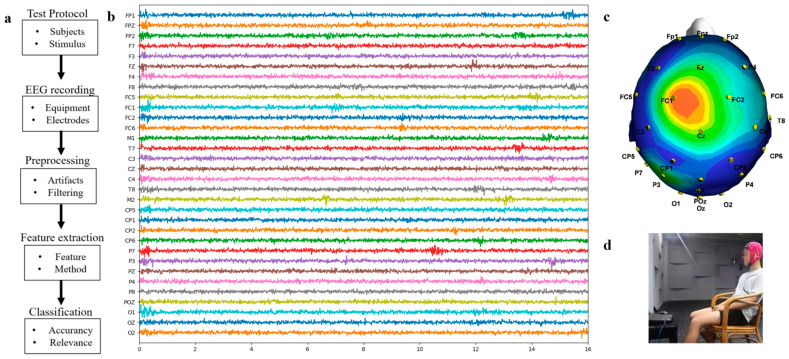
(**a**) The general flow of music state recognition; (**b**) recorded EEG responses (from the Subject 5 samples); (**c**) electrode and signal mapping relationship; (**d**) experimental collection environment.

The EEG signal acquisition device used in this study was produced by ANT-NEURO in Germany and is equipped with 32 electrode caps that adhere to the International 10–20 System standards set by the International Society for Electroencephalography. Figure 1a shows the overall process of EEG music composition state recognition. Figure 1b details the electrode layout corresponding to each signal. Figure 1c reveals the specific positions of the electrodes on the scalp by showing the EEG topography of subject 5 at a particular moment. To effectively isolate noise and electromagnetic interference, the collection of EEG data was conducted inside the school’s music recording studio, as shown in Figure 1d.

Data acquisition was conducted at a sampling rate of 200 Hz. Electrodes were attached to the scalp with conductive EEG paste to ensure reliable signal capture. The experiment was initiated only after verifying that the impedance of all electrodes had been reduced to below 2.5 kΩ.

#### 2.1.3. Experimental Paradigms and EEG Signal Acquisition

To focus on exploring the impact of musical melodies on the brain and to exclude the interference of emotions or other non-melodic factors, we collected electroencephalogram (EEG) data from subjects during the passive reception of musical stimuli and active recall and continuation of melodies. To ensure the emotional state of the subjects remained consistent throughout the experiment, we carefully selected music capable of eliciting specific emotions (happiness or sadness) as stimuli, avoiding music that could cause emotional fluctuations. Moreover, we assessed the impact of these musical stimuli on the subjects’ emotional states via a pre-experiment evaluation, ensuring the consistency of the subjects’ emotional states during different phases of the experiment (including the reception of musical stimuli and the process of recalling and continuing melodies). These measures aimed to minimize the interference of emotional variations on the experimental results, allowing us to more accurately assess the impact of musical melodies themselves on brain activity. The music information used in this experiment is shown in Table 2.

**Figure 2:**
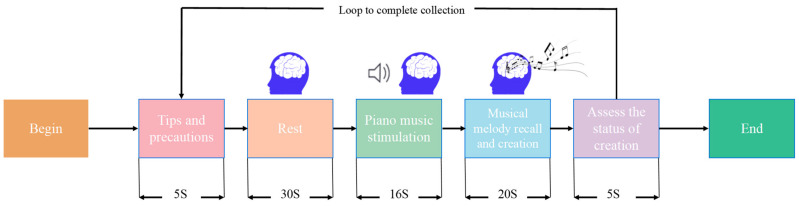
Music recall and continuation experimental paradigm.

This paper innovatively simulated the music creation process by having participants recall and extend melodies. This approach was inspired by the research of Halpern and Zatorre [24], who observed brain activity during musical imagery using functional magnetic resonance imaging (fMRI). They discovered that the brain regions activated during the imagination of melodies, particularly the temporal lobes, show similarities to those activated during actual music listening. These findings provide an experimental basis and inspiration for our attempt to simulate the state of music creation and explore the differences in brain activity between passive auditory stimulation and active creation. The specific experimental paradigm is illustrated in Figure 2.

Participants were presented with a piano solo consisting solely of the main melody, without accompaniment or chords, lasting 16 s. This was followed by a 20 to 30 s interval for them to recall and continue the melody, thereby simulating the music creation process. The piano melody was chosen by Music Conservatory professors experienced in arrangement and performance. Regarding the emotional content of the piano pieces, two prevalent emotions in music creation, happiness and sadness, were represented by four piano pieces each. After recalling and continuing the music, participants had the opportunity to assess their creative performance and, if dissatisfied, could opt to repeat the process. This approach was aimed at ensuring the quality of the collected data. The data from all participants were compiled and archived in EDF format to complete the dataset construction.

### 2.2. Proposed SSAM Network Framework

**Figure 3:**
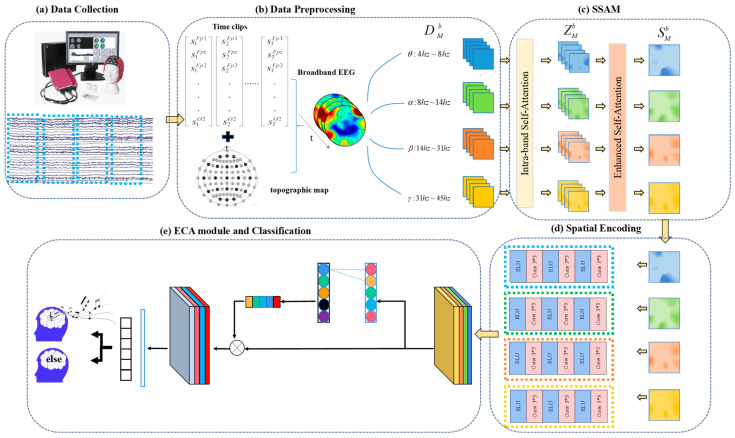
The overall framework of the proposed SSAM for identifying music creation status. (**a**) Data collection module. (**b**) Data preprocessing module. (**c**) SSAM self-attention network. (**d**) Spatial encoding module. (**e**) ECA and classification module.

The SSAM network is designed to capture essential information from brain areas across different frequency bands during high-level cognitive activities while minimizing interference from information unrelated to the targeted cognitive activity. This approach is inspired by fMRI and neuroscience research [23], which suggests that brain regions engaged in high-level cognitive activities, such as music creation or memory retrieval, tend to remain stable over extended durations. Additionally, information from various frequency bands exhibits distinct correlations with different cognitive processes. In light of these studies, a self-attention network based on the steady-state activation map (SSAM) framework has been developed. Its purpose is to investigate the similarities and differences between external music stimulation and internal music creation, integrate information across different frequency bands to identify brain regions most closely related to the state of music creation, and clarify the connectivity patterns among electrodes. The framework of the SSAMAN is shown in Figure 3.

In the SSAM model, the preprocessed, collected signals are first decomposed into four frequency bands most relevant to cognitive activities: the θ band (4–7 Hz), α band (8–13 Hz), β band (14–30 Hz), and γ band (31–45 Hz). A feature extraction module and a feature mapping module are then employed to convert the extracted feature sequences. This process transforms these sequences into a two-dimensional feature matrix sequence that is better suited for mining spatial information.

The self-attention network based on the SSAM first amplifies information from brain regions that are persistently or repeatedly activated. The network subsequently discerns the significance of various temporal map frames and integrates this information into a steady-state activation map. Three CNN layers are utilized to extract spatial information from this activation map. The ECA module subsequently captures prominent information from the information across different frequency bands of all electrodes, transforming these key features into the final attention representation. Finally, a SoftMax classifier maps the music creation-related information to the class label space, which is used to infer the current state.

### 2.3. Data Preprocessing Module

#### 2.3.1. EEG Signal Preprocessing

Raw EEG signals contain external noise and various artifacts (such as those from blinking or body movements), which can contaminate the data. Utilizing these raw, unprocessed signals directly in analyses can severely compromise the integrity of the experiments and the accuracy of the results. Therefore, preprocessing of EEG signals to remove these unwanted noises and artifacts is crucial before proceeding with further experimental analyses. As indicated in prior research, five salient EEG frequency bands lie within the 0–50 Hz range. The first frequency band δ, ranging from 0 to 4 Hz, is predominantly observed during deep sleep [25]. The subsequent four frequency bands θ, α, β, and γ, spanning 4–8 Hz, 8–13 Hz, 13–30 Hz, and 30–50 Hz, respectively, are intimately linked to cognitive activities in awake states [26]. Our study focuses on these latter four bands.

Signal preprocessing begins with the application of a third-order Butterworth bandpass filter to isolate the target frequency bands, effectively removing high-frequency noise. This step is followed by the use of a notch filter to specifically eliminate power line noise at 50 Hz, further purifying the signal. Visual inspection of the signals is then conducted to identify and eliminate intrinsic artifacts, such as eye blinks (EOG) and muscle movements (EMG), which could potentially bias the analysis results. Finally, spatial filtering based on Independent Component Analysis (ICA) is employed to remove any remaining sources of noise.

In the preprocessing phase, data from eight participants were excluded due to significant signal fluctuations or abnormalities, such as excessive blinking or involuntary movement, which could skew the analysis. This exclusion criteria aimed to ensure the highest fidelity in identifying the brain regions and electrodes most relevant to the music creation process. The data ultimately fed into the model are shown in Table 3.

#### 2.3.2. Feature Topographic Matrix Mapping

After preprocessing the data, four bandpass filters were applied to extract EEG signals in the θ, α, β, and γ frequency bands. Differential entropy (DE) was then utilized to extract features from these signals. As a time–frequency domain feature proposed by Duan et al. [27], DE has been proven to be extremely effective in cognitive neuroscience research involving EEG signals. Considering that EEG signals can be viewed as finite-length signals adhering to a Gaussian distribution $N(\mu ,\sigma^{2})$, we apply a well-established formula for differential entropy (DE), which has been simplified from its original definition for practical use in this context. This results in the following expression:

(1) $$DE(x)=-\int_{-\infty }^{\infty }p(x)\log(p(x))dx=\int_{-\infty }^{\infty }(\frac{1}{\sqrt{2\pi \sigma^{2}}}e^{-\frac{{\left(x-\mu \right)}^{2}}{2\sigma^{2}}})\log(\frac{1}{\sqrt{2\pi \sigma^{2}}}e^{-\frac{{\left(x-\mu \right)}^{2}}{2\sigma^{2}}})dx=\frac{1}{2}\log(2\pi e\sigma^{2})$$

where μ and σ represent the mean and the standard deviation of the EEG signals, respectively; p(x) represents the probability density function of the EEG signal; and e represents Euler’s constant.

The mapping of the feature topographic map is depicted in the figure below. A 1 s nonoverlapping time window is introduced for the preprocessed EEG signal. Based on the electrode placement of the EEG cap’s 10–20 system [28], signal features from each time window are mapped into a 9 × 9 two-dimensional matrix. The use of nonoverlapping time windows aims to prevent any interference with the performance of the subsequent SSAM attention network, specifically by preventing redundant amplification or attenuation of information from areas where time windows might overlap.

**Figure 4:**
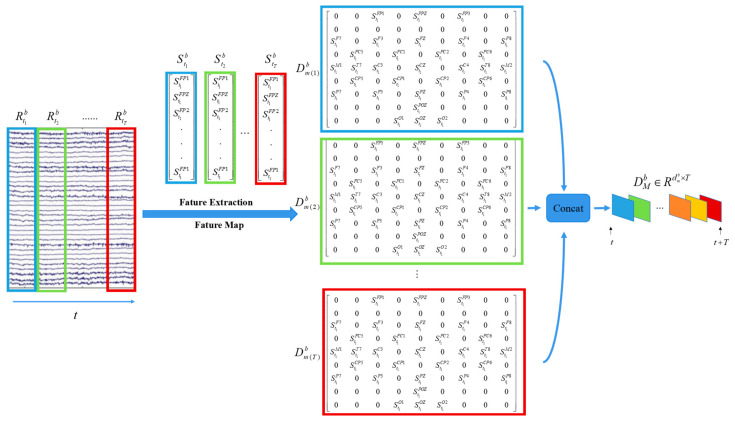
Mapping process of EEG signals to the DE feature topography map.

Figure 4 shows the entire feature extraction and feature map module. The raw EEG signals are represented as $R_{t_{x}}^{b}\in R^{C\times T\times f}$, where $b\in \left\{\theta ,\alpha ,\beta ,\gamma \right\}$, *C* equals the number of electrodes (32), *T* is the total duration (16 s), and *f* is the sampling frequency (128 Hz). The signals are then sliced into 16 one-second nonoverlapping clips for subsequent feature extraction. In each clip, Equation (1) is used to extract DE features in different bands, yielding a set of DE feature vectors, denoted as $S_{t_{x}}^{b}=[s_{t_{x}}^{FP1},s_{t_{x}}^{FPZ},\cdots ,s_{t_{x}}^{O2}]\in R^{b\times C\times t_{x}}$, where $t_{x}$ represents the clip number. The time series of $t_{x}$ seconds are mapped into a 9 × 9 matrix according to the EEG cap arrangement shown in Figure 4. Finally, the feature matrices of the same frequency band are concatenated to form the input of the entire model, represented as $D_{M}^{b}\in R^{d_{m}^{b}\times T}$, where M={m(1),m(2),…,m(T)} is a collection of feature matrices, and $d_{m}^{b}=9\times 9$ indicates the size of the feature matrix at a certain time $t_{x}$ under a certain frequency band *b*.

### 2.4. Steady-State Activation Mapping (SSAM) Module

**Figure 5:**
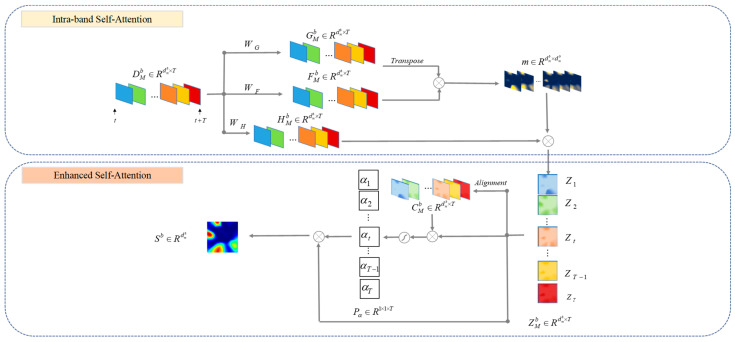
The details of the intra-frequency band self-attention mechanism used in the method.

Subsequent to the feature topographic mapping module, SSAM is utilized to identify and amplify the information within the music-relevant brain regions. As illustrated in Figure 5, the SSAM is composed of two layers of attention networks: the attention network between feature time matrix sequences and the extended self-attention network. These two networks are structured to compute the steady-state activation map for the music recall creation state from $D_{M}^{b}\in R^{d_{m}^{b}\times T}$ in the current frequency band.

#### 2.4.1. Submodule: Intra-Band Self-Attention

The attention network aims to analyze the dynamic responses of specific brain areas to musical melodies over time. It scrutinizes sequences of data across various time frames to pinpoint regions that actively respond to music within the selected frequency band. By doing so, the network not only highlights the information from regions sensitive to musical stimuli but also diminishes the influence of irrelevant areas. This selective enhancement and suppression process refines our insight into how music influences brain activity, emphasizing the contributions of the most responsive regions. This approach assists in minimizing the disturbance caused by the brain’s handling of irrelevant information, enabling a focused analysis of information associated with the music recall creation state.

The specific calculation steps are as follows: Initially, the module maps the feature matrix sequence $D_{M}^{b}$ into the feature spaces *G* and *F* via two feature transformations, $g^{b}$ and $f^{B}$. This is achieved by utilizing two 1×1×C convolution kernels.

(2) $$\left\{\begin{array}{l} G_{m(t)}^{b}=g^{b}(D_{m(t)}^{b})=W_{G}D_{m(t)}^{b}\\ F_{m(t)}^{b}=f^{b}(D_{m(t)}^{b})=W_{F}D_{m(t)}^{b} \end{array}\right.$$

In this formula, $G_{m(t)}^{b},F_{m(t)}^{b}\in R^{d_{m}^{b}}$. The matrices *G* and *M* are utilized to search for and discern information pertinent to the music recall state, capturing the intricate dependencies among feature matrices at disparate moments.

To ascertain the importance of the feature matrix at each moment within the entire sequence of time frames, the SoftMax function was applied. This method calculates the probabilities indicating the significance of each moment’s feature matrix in relation to the entire sequence, where higher values highlight moments that are more relevant to the analyzed state:

(3) $$m{\left(t\right)}_{\left(p,q)(i,j\right)}^{b}=\frac{\exp{\left(G_{m{\left(t\right)}_{\left(i,j\right)}}^{b}\right)}^{T}(F_{m{\left(t\right)}_{\left(p,q\right)}}^{b})}{\sum_{m,n}\exp{\left(G_{m{\left(t\right)}_{\left(i,j\right)}}^{b}\right)}^{T}(F_{m{\left(t\right)}_{\left(m,n\right)}}^{b})}$$

Here, $G_{m{\left(t\right)}_{\left(i,j\right)}}^{b}$ denotes the position (i,j) in feature map *G* at the *t*-th time under frequency band *b* and likewise represents the position (p,q) in feature map *F* at the *t*-th time under frequency band *b*. $m{\left(t\right)}_{\left(p,q)(i,j\right)}^{b}\in R^{d_{m}^{b}\times d_{m}^{b}}$ signifies the influence or contribution of position (i,j) in feature map *G* to position (p,q) in feature map *F*. In other words, this value represents the model’s degree of attention to information in different brain areas. A high degree of attention suggests that the information in this area is strongly correlated with the task status.

In this step, the feature representation at each position of the original feature map $D_{M}^{b}$ is recalibrated and weighted using the previously computed attention weight $m{\left(t\right)}_{\left(p,q)(i,j\right)}^{b}$.

(4) $$Z_{m{\left(t\right)}_{\left(i,j\right)}}^{b}={\sum_{p,q}m(t)}_{\left(p,q)(i,j\right)}^{b}\times D_{m(t)(p,q)}^{b}$$

The purpose of this step is feature recalibration, which involves enhancing or weakening the information at each feature location contingent upon its correlation with all other feature locations. In particular, the model amplifies the weight of information in task-related brain regions and mitigates the impact of information in irrelevant regions based on the level of activation. This recalibration allows the model to more effectively discern dependencies among different feature locations, thus facilitating the learning and comprehension of more complex and abstract visual patterns. Then, $Z_{M}^{b}\in R^{d_{m}^{b}\times T}$ is input into the extended self-attention network after feature calibration.

#### 2.4.2. Submodule: Enhanced Self-Attention

To extract more discriminative state information, integrating task-related information across various time series is essential. An extended self-attention mechanism is employed to reassign weights to these matrices, enabling the probing of the intrinsic significance of feature matrices at different times. Distinct from traditional self-attention mechanisms [29], the extended attention mechanism serves as a natural expansion of additive attention at the multidimensional feature level. This implies that the extended attention mechanism processes attention to higher-dimensional features and is adept at capturing and illustrating the intricate structures and relationships inherent in the data.

The extended self-attention network is designed to assign varying degrees of importance to the enhanced feature time matrix sequences received from the aforementioned attention network and to integrate this information to construct the final steady-state activation map. This approach reveals the brain areas and electrodes most strongly correlated with the creative state of music recall in this frequency band.

The first step is to reveal the intrinsic importance of different time series, which reflects their correlation with the target cognitive activity. Initially, the alignment pattern vector $C_{M}^{b}$ of $Z_{M}^{b}$ is obtained via a linear transformation. Subsequently, the information score contained in the feature matrix of each frame is calculated using the following equation:

(5) $$I_{m(t)}^{b}=f(Z_{m(t)}^{b},C_{m(t)}^{b})=W_{3}\sigma (W_{2}Z_{m(t)}^{b}+W_{1}C_{m(t)}^{b}+b_{2})+b_{1}$$

In the given formula, $f(Z_{m(t)}^{b},C_{m(t)}^{b})$ indicates the inherent convergence of the internal working mode within brain regions during the state of music recall and creation. This implies that the attention model is more attuned to brain areas operating under the same or similar modes, namely those active in the music recall and creation state. Within the formula, the activation function σ(⋅) is an exponential linear unit. *W*_1_, *W*_2_, and *W*_3_ are weight parameters, while *b_1_* and *b_2_* serve as bias terms. Subsequently, the feature matrix of each frame depicts the magnitude of task-related information. Based on this information, the model determines the significance of each frame throughout the entire process. Thus, the importance of the *i*-th feature matrix within the overall state process can be expressed as follows:

(6) $$\alpha_{i}=\frac{e^{{\left(I_{m(t)}^{b}\right)}^{T}(Z_{m(t)}^{b})}}{\sum_{t=0}^{T}e^{{\left(I_{m(t)}^{b}\right)}^{T}(Z_{m(t)}^{b})}}$$

Finally, information from different time points is integrated to produce the final steady-state activation map. Throughout its feature learning process, the SSAM network incrementally mines and accentuates the input information associated with music recall and creation, consolidating spatial–temporal information across the entire state. The calculation formula for the SSAM is as follows:

(7) $$S^{b}=\sum_{i=0}^{T}P_{\alpha (i)}^{b}Z_{m(i)}^{b}$$

### 2.5. CNN Spatial Encoding Module

The SSAM module is structured to amplify information associated with the music recall state, while the primary function of the CNN module is to locate and extract this information. To prevent the intermixing of information across frequency bands, one CNN layer is exclusively applied to each specific frequency band, ensuring the preservation of the unique information of certain bands.

The input to the CNN module consists of EEG feature vectors $S^{b}\in R^{d_{m}^{b}}$ previously computed by the SSAM module, with each vector corresponding to a specific frequency band $b\in \left\{\theta ,\alpha ,\beta ,\gamma \right\}$. For these four frequency bands, we designed a separate CNN layer for each, aimed at precisely capturing task-related information contained within each band. To achieve this, the CNN architecture for each band is composed of three convolutional layers, all utilizing 3 × 3 convolutional kernels to optimize computational efficiency and reduce the number of model parameters. The layers are equipped with 32, 64, and 128 filters, respectively, with an increasing number of filters at each layer to help the model capture richer feature representations at various levels of granularity. The choice of 3 × 3 convolutional kernels is based on their ability to reduce the number of parameters and computational load while increasing the depth of the network to enhance the feature abstraction capability and the generalization performance of the model [22]. After convolutional layer processing, the Scaled Exponential Linear Unit (SELU) is adopted as the activation function to address gradient vanishing and explosion, outperforming the traditional ReLU [30]. By normalizing activations, SELUs improve the network’s stability and adaptive learning.

After the above processing, the CNN module transforms the feature vectors of each frequency band into advanced feature representations rich in deep abstract information $Y^{b}=conv(SSAM^{b})$, where $Y^{b}\in R^{d_{m}^{b}\times c}$ and *c* is the number of filters in the convolutional layer. These advanced representations are highly valuable to the model as they enhance its ability to interpret and classify complex patterns in EEG signals, which is extremely beneficial for improving the accuracy of EEG data processing.

### 2.6. ECA Module and Classification

After the convolutional layer extracts high-dimensional feature representations, the ECA module is integrated to delve deeply into the frequency features of differently encoded EEG sequences. This approach allows us to more precisely extract distinguishing information from EEG features across each frequency band.

**Figure 6:**
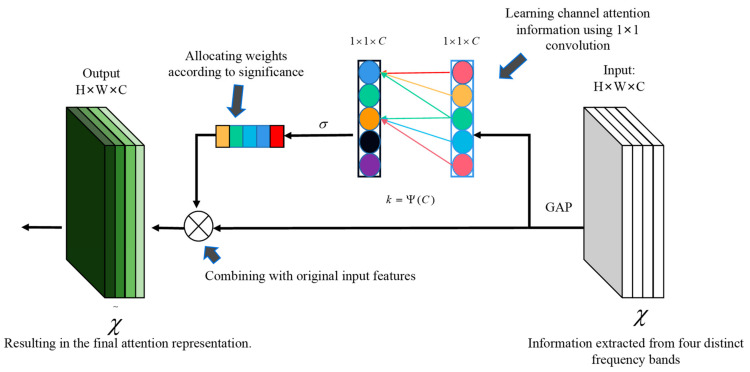
Details of the ECA module used in the model.

The ECA network pivots around channel attention, deviating from conventional spatial attention. Its principal mechanism calculates inter-channel correlations to determine attention weights. This approach accentuates pivotal feature channels and diminishes less crucial ones. Compared to traditional attention mechanisms, the ECA module offers superior efficiency in terms of parameters and computations, bypassing additional parameters and intricate spatial procedures. The number of convolution kernels *k* of the ECA module is determined by the following formula:

For the high-dimensional outputs $Y^{\theta },Y^{\alpha },Y^{\beta },Y^{\gamma }\in R^{d_{m}^{b}\times c}$ from the convolutional layer, the ECA module begins with global average pooling, followed by the use of *k* convolution kernels to discern dependencies between channels. This process adjusts features based on these insights, culminating in the final feature representation. The formula for determining *k* and the total number of channels *C* is as follows:

(8) $$k=\Psi (C)=|\frac{\log_{2}(C)}{\lambda }+\frac{b}{\lambda }|$$

where λ and *b* are hyperparameters; in this paper, they are set to 2 and 1, respectively. With these parameters, the model achieves good results. The workflow of the ECA module is depicted in Figure 6. The ECA module is specifically designed to accentuate pivotal frequency band features while minimizing irrelevant features, thereby enhancing the accuracy and resilience of the model.

After flattening the final feature representation, the fully connected layer maps all the feature vectors to a one-dimensional vector $X_{raw}$. During the training process, a dropout layer is utilized to enhance the model’s generalization ability, with the dropout rate set between 0.3 and 0.5. This produces the input for the recognition module, denoted as $X_{drop}$.

Following the ECA module, the classification task of the model is to distinguish between two distinct electroencephalogram (EEG) states: the external musical stimulation state and the internal music recall and creation state. This binary classification task is crucial for understanding how music affects brain activity. Once the feature representation is fed into the identification module, the SoftMax function is responsible for calculating the conditional probabilities for each state category. For each input sample, SoftMax outputs the category with the highest conditional probability as the prediction outcome. This process ensures that the model can accurately differentiate between the two given state categories, thereby fulfilling the classification task between musical stimulation and recollection states. The computational process is detailed as follows:

(9) $$y=argmax_{i}P(y=i|x)=argmax[softmax(W_{d}X_{drop}+b_{d})]$$

where $W_{d}$ and $b_{d}$ are the parameters for the identification module. The loss function is the negative log-likelihood for the category $y_{i}$, with L2 regularization applied. The formula is as follows:

(10) $$Lost(\theta )=-c_{i}\log(y_{i})+\lambda {\left\|\theta \right\|}_{2}^{2}$$

In the equation, $c_{i}$ represents the one-hot encoding of label $y_{i}$, while θ denotes the model’s initial state or position. The optimizer used is Adam. The model calculates the current loss function Lost(θ) based on the current value of θ, computes the gradient, and adjusts the parameters until the loss function meets a specific threshold. This step can ensure optimal performance in the classification task of music stimulation and recall and creation states.

## 3. Results

This section comprehensively presents the experimental evaluation of the proposed model on a self-constructed dataset, including the experimental setup, the experiments conducted, and the analysis of the results. Additionally, it reveals the activation of brain regions and their collaborative patterns during states of musical recall and creation.

### 3.1. Implementation Details

In EEG recognition tasks, experiments are commonly conducted in either a subject-dependent or subject-independent manner. Given the highly subjective nature of music recall and creation, which can be influenced by individual personality, education level, experience, and other factors, our model adopts a subject-dependent training approach [31]. For this experiment, we train a separate model for each participant and employ ten-fold cross-validation to determine each subject’s accuracy. The final recognition rate of the model is the average classification accuracy across all subjects.

The initial learning rate of the model is set at $10^{-4}$. When the recognition rate reaches 70%, it is reduced to $5\times 10^{-5}$ and further adjusted to $10^{-5}$ upon reaching an 85% accuracy rate. Each subject’s model undergoes 50 iterative updates with a batch size of 32. This batch size can be adjusted based on the training conditions of individual subjects, with the majority achieving optimal results at batch sizes of 32 or 64. In addition to using the overall recognition rate as an evaluation criterion, this study also analyses the recognition rate for each state and the contribution rate of each frequency band, aiming to comprehensively evaluate the classification performance and effectiveness of our model from multiple perspectives.

### 3.2. Performance Comparison with Related Works

This study compares the proposed method with established approaches from public datasets and evaluates its performance using a specially designed dataset, with detailed results presented in Table 4. The model exhibits a substantial improvement in the accurate differentiation of cognitive states elicited by external musical stimuli and those arising from internally recalled creative processes across both happy and sad emotional music contexts. For untrained participants, accuracy improved by 1.55% for cognitive states related to happy music and by 4.69% for sad music. Participants with a musical background showed improvements of 1.39% for happy music-related states and 2.96% for sad music-related states. Significantly, the model achieved considerable performance gains in cognitive states associated with sad music, a scenario where most models typically exhibit lower recognition rates. These findings highlight the effectiveness of our approach, which prioritizes the extraction of information from highly active brain regions in response to specific emotional music stimuli and selectively disregards less pertinent data, thereby facilitating more effective processing of complex cognitive activities.

A confusion matrix is employed to create the other metrics frequently from the fundamental metrics (true positive/TP, true negative/TN, false positive/FP, and false negative/FN). The number of correct predictions to the total number of predictions yielding the quality metric is called classification accuracy (ACC). However, ACC is not accepted as an adequate parameter to determine the proposed algorithm’s performance totally. Hence, the other statistical performance metrics are described, including precision and specificity in this respect. The notations are given below.

(11) $$ACC=\frac{TP+TN}{TP+FP+FN+TN}$$

(12) $$Precision=\frac{TP}{TP+FP}$$

(13) $$Specificity=\frac{TN}{FP+TN}$$

Based on these metrics, we recalculated the performance of each method, as shown in the Table 5 below.

**Figure 7:**
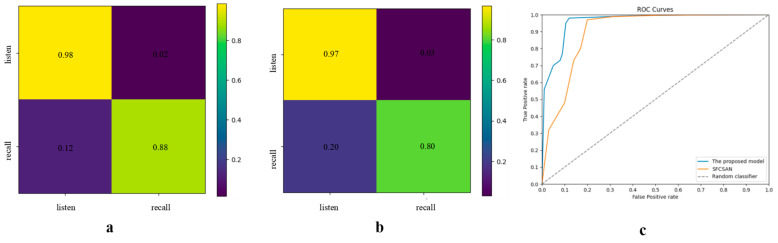
Model performance. (**a**) Confusion matrices of the proposed model; (**b**) confusion matrices of the best-performing comparison model, SFCSAN; (**c**) Roc curve of two methods.

To precisely analyze the performance of this model in differentiating various states compared to the best-performing model in the group, SFCSAN. Confusion matrices and ROC curves are established, and the detailed results are shown in Figure 7. Although both models exhibit similar performance in recognizing music stimulation states, the proposed model achieves an 8% improvement in distinguishing between music recall and creation states. The results of the ROC curve also show the superiority of our method. This result highlights the model’s advantage in extracting distinctive and relevant information associated with music recall and creation states. Further analysis of this information could reveal potential mechanisms of brain activity during the music creation process.

### 3.3. Visualization of the SSAM Network

**Figure 8:**
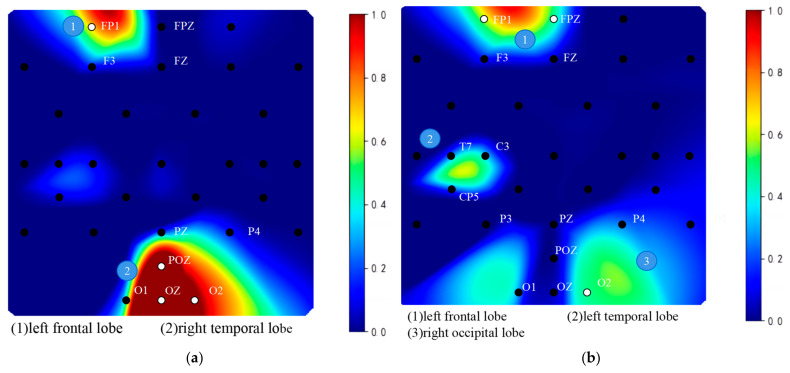

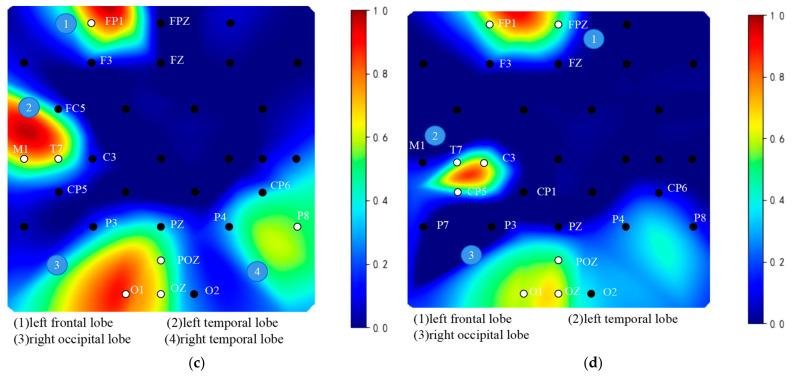
Attention maps of the SSAM model. (**a**) Brain regions activated during the creative process under the happy emotion condition for subjects without training. (**b**) Brain regions activated during the creative process under the sad emotion condition for subjects without training. (**c**) Brain regions activated during the creative process under the happy emotion condition for musically trained subjects. (**d**) Brain regions activated during the creative process under the sad emotion condition for musically trained subjects.

The proposed model excels at extracting discriminative information, specifically targeting brain regions rich in music-related content. Electrodes associated with these regions are posited to contain a greater abundance of music-related information, which is instrumental for correlating brain electrical activity with music. Analysis of the attention map from the SSAM attention network involved setting a threshold and visualization, yielding the following results in Figure 8.

The attention map shows that the brain regions most associated with music recall and creation are predominantly on the left side, particularly concentrated in the left prefrontal lobe (FP1 and FPZ), left temporal lobe (T7), and occipital lobe (O1 and OZ). When comparing subjects without musical training to professionally trained subjects, the left temporal lobes (M1 and C3) in the latter group exhibited heightened activation. Moreover, additional activation was observed in the right occipital lobe (O2 and P8). The behavior of these regions likely stems from the shaping influence of music on the brain. Elucidation of these areas is hoped to offer valuable insights and support for EEG music generation.

**Figure 9:**
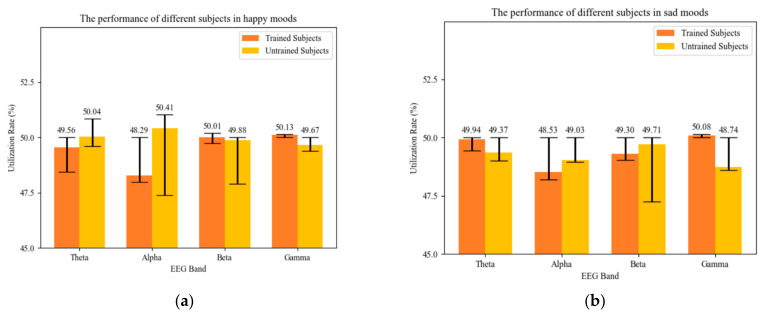
Contribution of the four frequency bands to the recognition rate of the creative state in different emotions. (**a**) Performance of different subjects in happy moods. (**b**) Performance of different subjects in sad moods.

Figure 9 shows the contributions of each of the four frequency band outputs from the ECA module. Individuals without musical training predominantly utilize EEG information in the alpha band. In contrast, those with musical training have more information related specifically to music creation in the gamma band. The contributions from the beta and theta bands did not significantly differ. A comparison of the differences in recognition rates between the two datasets suggested that the high-frequency gamma-band contains more relevant information than the low-frequency alpha band for music-related complex cognitive activities, indicating the need for greater emphasis on information within this frequency range.

### 3.4. Activation Analysis of Brain Regions in Music-Related Cognitive Activities

The focus of this section is the activation analysis of brain regions during music-related cognitive activities, specifically examining whether the methods typically used in emotion recognition are applicable to more complex cognitive tasks such as music recall and creation. In emotion recognition studies, researchers often employ a time-slicing method with overlapping EEG windows, typically ranging from 1 to 3 s [40,41,42], to segment EEG signals. This approach not only expands the sample size but also allows deep learning algorithms to extract richer information, achieving commendable results in emotion recognition studies. However, the suitability of this method for more complex cognitive activities [43,44], such as those involved in musical creativity, requires further experimental validation. The present study selected individuals with the highest recognition rates from two groups of subjects. After preprocessing the original EEG signal, a two-second window was used for calculating the feature matrix sequence. Compared to a one-second window, the data from the two-second window were smoother and less influenced by transient stimuli. The correlation of these data with the SSAM matrix generated by the proposed model was calculated, setting a threshold of 0.6 to identify relevant information. Locations with a correlation coefficient below this threshold were considered to contain less information pertinent to the music recall and creation states.

**Figure 10:**
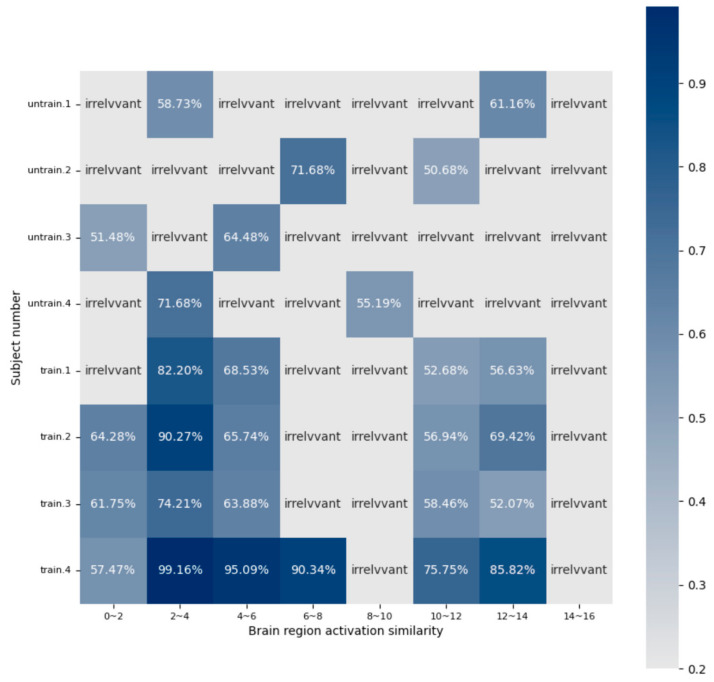
Correlation between the attention matrices obtained by SSAM and the feature matrices of different time segments. We selected data from the eight subjects with the highest recognition rates for analysis. Correlations lower than 0.5 were deemed unrelated.

The heat map presented in Figure 10 illustrates that, regardless of the emotional context, the characteristic patterns of brain activity associated with the states of music recall and creation are not continuously active but exhibit intermittent activation. This suggests that the brain regions involved in processing music-related tasks are not persistently active but are instead subject to frequent activations over brief intervals. This phenomenon is more pronounced in participants without musical training. In contrast, individuals with a musical background, particularly those with high recognition rates, exhibit shorter activation intervals and stronger activation intensity during music recall and creation states, suggesting that musical training may enhance brain activity and creativity.

Furthermore, the heatmap results suggest that while the time-slicing technique is effective at capturing emotion-related information, it may have limitations in identifying the subtle nuances of more complex cognitive activities, such as music composition. This finding underscores the advantage of the proposed method in capturing relevant information across different time frames in complex cognitive tasks.

### 3.5. Network Connectivity Analysis in Music-Related Cognitive Activities

**Figure 11:**
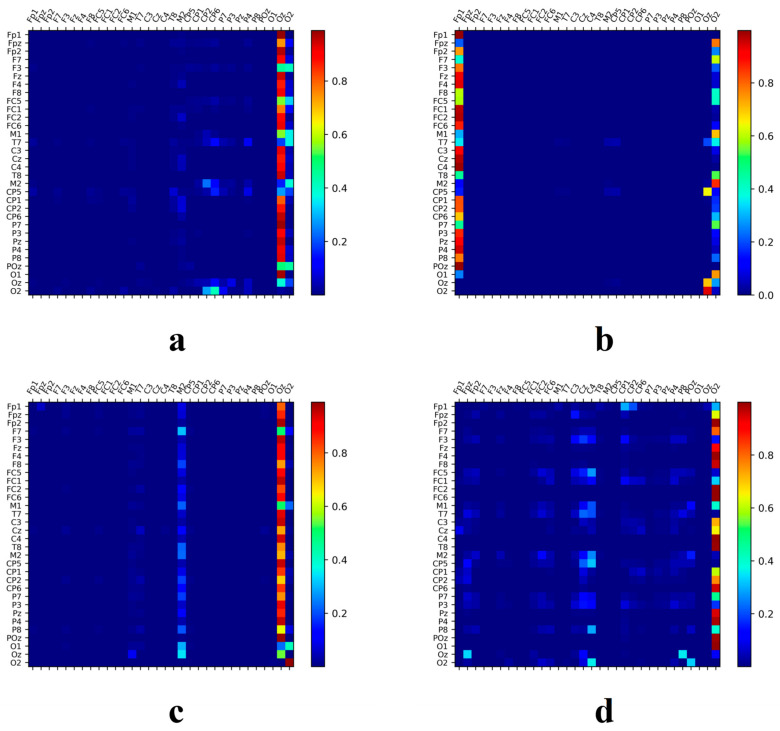
The neural activation pattern of untrained subjects under happy musical stimulation. (**a**) Band θ; (**b**) Band α; (**c**) Band β; (**d**) Band γ.

**Figure 12:**
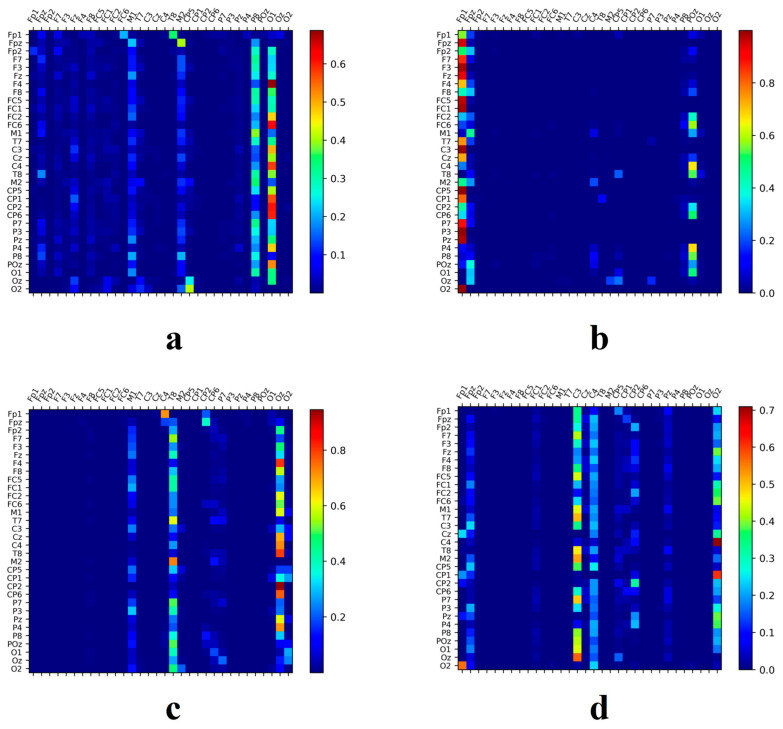
The neural activation pattern of trained subjects under happy musical stimulation. (**a**) Band θ; (**b**) Band α; (**c**) Band β; (**d**) Band γ.

**Figure 13:**
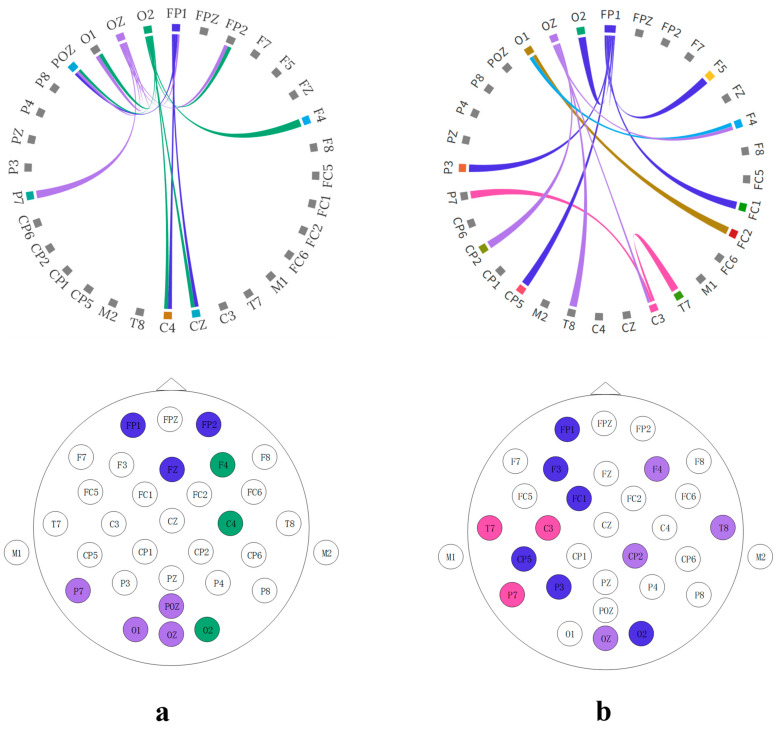
Comparison of brain connectivity among subjects with different training levels under happy musical stimulation: (**a**) untrained subjects; (**b**) trained subjects.

Using the SSAM network, the electrodes most closely associated with music recall and creation states were identified. Additionally, this paper further investigated how other brain regions work in tandem with these identified electrode areas. To this end, this study explored the correlation of temporal feature sequences among different electrodes, seeking to discern collaborative patterns within these regions. The heatmap matrices depicted in Figure 11 and Figure 12 showcase the working synergy between the brain areas corresponding to these electrodes. Circle plots based on set thresholds further reveal the functional connections that are most relevant to these key areas. The results are presented in Figure 13.

The analysis indicates a significant correlation between electrodes closely related to music recall and creation and other electrodes. The heatmap matrix reveals that individuals trained in music exhibit brain activation levels significantly greater than those reflected by the SSAM network, even in specific regions that may not contain much state-specific information.

During happy emotions, individuals without musical training predominantly display synchronized brain activity in the frontal (FP1 and FP2), vertex (CZ), left occipital (O1, O2, and POZ), right temporal (F4 and C4), and right occipital (O2) regions, with the central brain region showing less obvious activation in higher frequency bands. In contrast, those with musical training show more intense synergistic activity in the left frontal (FP1 and F3) and left temporal (CP5 and P3) regions, vertex area (CP2), and right temporal area (F4 and T8), demonstrating pronounced collaboration with key brain regions. The activation in the parietal and temporal regions of these individuals is notably extensive.

**Figure 14:**
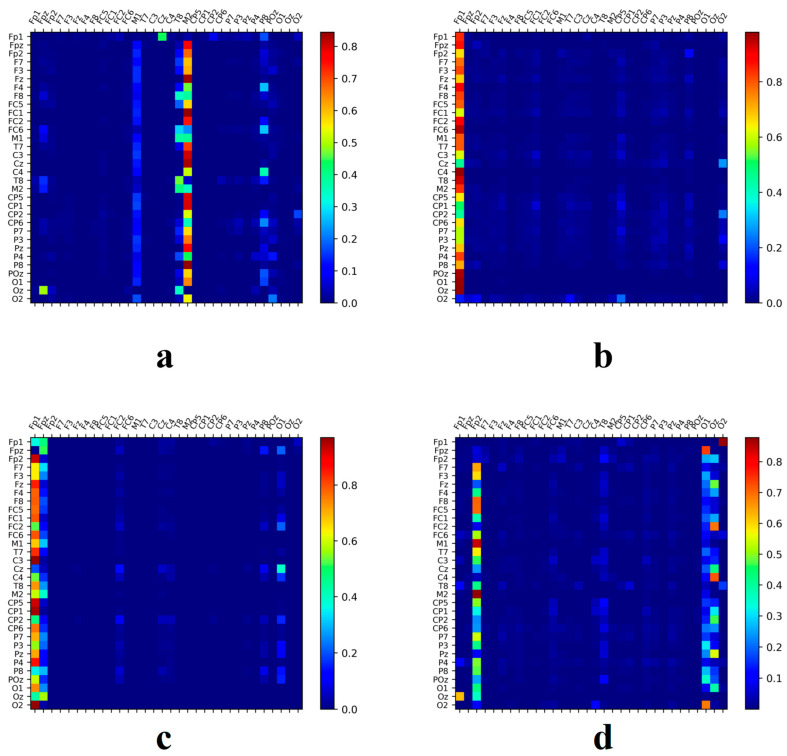
The neural activation pattern of untrained subjects under sad musical stimulation. (**a**) Band θ; (**b**) Band α; (**c**) Band β; (**d**) Band γ.

**Figure 15:**
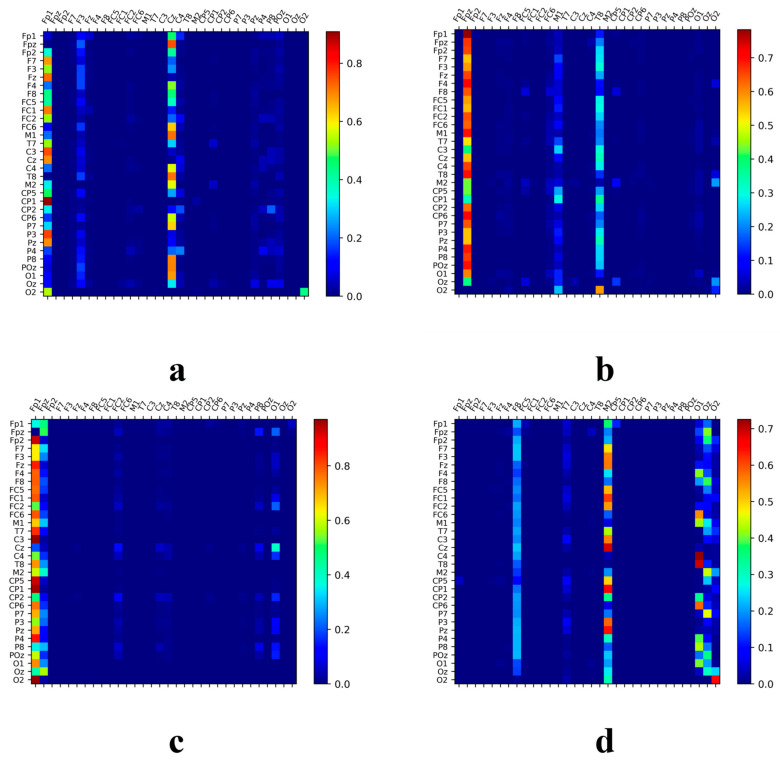
The neural activation pattern of trained subjects under sad musical stimulation. (**a**) Band θ; (**b**) Band α; (**c**) Band β; (**d**) Band γ.

**Figure 16:**
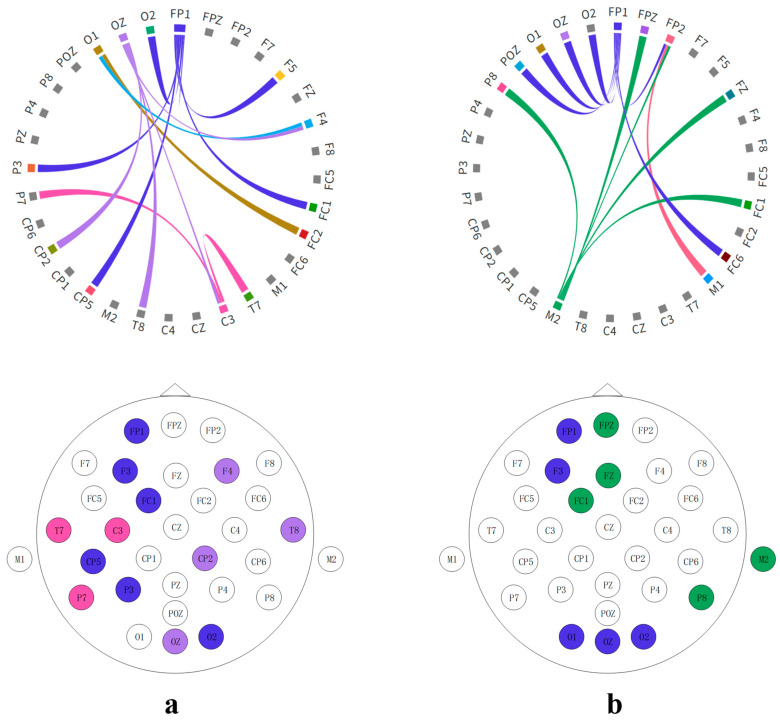
Comparison of brain connectivity among subjects with different training levels under sad musical stimulation: (**a**) untrained subjects; (**b**) trained subjects.

As shown in Figure 14, Figure 15 and Figure 16, in sad emotional states, both professionally trained and untrained participants exhibit noticeable activation in the prefrontal and right occipital cortices.

Participants without musical training demonstrate a relatively simple brain cooperation pattern, with only a few electrodes showing significant correlations with surrounding regions. Notably, the left frontal (FP1 and F3) and occipital (O1, O2, and OZ) regions, as well as the vertex (FZ and FC1) and right temporal areas (P8 and M2), exhibit strong synergistic activity. In contrast, individuals with musical training exhibit a more complex pattern of brain cooperation. While the correlations between electrodes are not as strong as those between electrodes of untrained participants, the involved areas are more extensive. The frontal (FP2), occipital (POZ and O2), and temporal (M1 and T8) regions show signs of collaboration. This finding suggests that musical training may reshape brain structures. Untrained individuals rely on specific brain regions to process music information, where these regions are closely interconnected. Conversely, musical training expands the brain’s processing areas, enabling different regions to handle various musical elements, thereby increasing processing efficiency. This pattern is confirmed by the higher recognition rates observed in the dataset, further emphasizing the plasticity of the brain and the profound impact of music on it. These findings could help elucidate the complex relationship between the brain and music, particularly in understanding the potential mechanisms of how electroencephalography can be transformed into musical compositions [45].

In light of the relatively small sample size, we performed a rigorous significance analysis to ensure the robustness of our findings, applying a *p* < 0.05 threshold for statistical relevance. The outcomes of this analysis are displayed across two tables: Table 6 and Table 7. Electrodes exhibiting significant correlations under various emotional music stimuli were predominantly localized in the left frontal (FP1 and FPZ) and occipital regions (O1, OZ, and O2). Notably, Table 6 reveals that in untrained participants, electrodes with higher significance were concentrated in the α band, whereas Table 7 indicates that, in participants with musical training, significant electrode activity was primarily observed in the γ band. These patterns suggest a differential neural processing response to musical training, highlighting potential areas of interest for further cognitive and neurophysiological research.

## 4. Discussion

In this section, we compare our research findings with the conclusions of existing literature. Although there has been no study focusing on the identification of music composition states like ours, there is substantial literature on the connection between music and the brain. The following is a revised discussion of our results from four aspects:

- Studies have shown that rhythm processing is primarily associated with the brain’s motor cortex (located in the frontal lobe) and the cerebellum. These areas are involved not only in motor control but also in the generation of rhythmic sense and the cognitive processing of rhythmic patterns [1]. This also explains why we observed activation in the frontal lobe likely associated with musical rhythm, leading to sustained activation of the frontal lobe due to the sense of rhythm in music.
- The temporal lobe plays a central role in processing the emotions, memories, and familiarity generated by music [46]. It was anticipated that familiar songs would trigger strong activation in the brain’s limbic and reward system areas, thereby producing pleasant emotions in musical auditions. In our experimental results, subjects with musical training showed significant activation in the temporal lobe, highlighting its core role in processing the emotions, memories, and familiarity generated by music.
- The occipital lobe’s association with cognitive neural activity mainly involves visual processing, including the processing and interpretation of signals received from the eyes [47,48]. Additionally, the occipital lobe is also associated with processing sensory inputs from sources other than the eyes, especially in blind individuals, where occipital lobe activity remains very active when using other senses (such as hearing, touch, and smell). This neural plasticity demonstrates the brain’s ability to adapt to different situations. Our paradigm involved piano music with no visual stimuli. Although the direct connection to music might not be as apparent as in other brain areas, it is still worth further exploration.
- In our study, the results of the ECA module revealed the importance of high-frequency bands (beta and gamma) in distinguishing music composition states, similar to findings in emotional recognition research [45,49,50], where high-frequency bands performed slightly better than low-frequency bands (theta and alpha). This result emphasizes the close association between music and emotions, as well as the significant driving role of emotions in music composition and experience.

## 5. Conclusions

In this research, a novel SSAM model was developed to identify EEG-related states of musical recall and composition. The approach began by enhancing information from brain regions that are continuously or repetitively activated via an attention network. An extended self-attention network was then utilized to integrate temporal data, resulting in the generation of a steady-state activation map. Spatial information within these activation maps was captured using a three-layer convolutional neural network (CNN). Complementary information between different frequency bands was extracted via the ECA network, leading to the formation of the final feature representation. Evaluation on a specifically constructed dataset revealed the model’s significant advantages in recognizing music recall and composition states: (1) performance improvements of at least 2.94% and 7.65% were observed in untrained subjects and subjects with a musical background, respectively; (2) the frontal (FP1 and FPZ) and occipital lobes (O1, O2, and OZ) were specifically identified as the brain regions most closely associated with music recall and creation states; (3) activation patterns in these brain regions are intermittent during these states; and, (4) additionally, collaborative relationships between different brain regions were explored via visualization analysis. We believe that applying brain regions related to music composition along with electrodes to brainwave music generation offers valuable guidance for exploring the connection between EEG characteristics and musical features, ultimately leading to the creation of superior music.

Despite the small sample size, our experiment not only achieved a high state recognition rate but also identified electrodes with statistically significant features, thereby proving the effectiveness of our method. The deep learning model we propose is capable of learning more distinctive information from a larger dataset, making it applicable even with more samples. Furthermore, although this method was primarily designed for musical recall and composition, it can also be applied to other complex higher cognitive activities. This is because we did not make any music-specific adjustments, but rather enhanced state-related information based on the activation of brain regions. This approach is applicable to any cognitive neural activity that can stably activate certain brain regions, capturing the brain areas and electrodes most relevant to that state.

### Future Work

(1). The main limitations of this study are the relatively small sample sizes that could lead to limited generalization of the findings. We will continue to expand the sample size and variety (including age and stimuli) to further validate our conclusions. Additionally, we will apply our model to other cognitive activities to verify its universality.

(2). In the current study, the musical stimuli employed were chordless piano pieces, which did not encompass other subtle elements of music, such as pitch, rhythm, and tempo. This selection may have constrained our understanding of music’s comprehensive impact, particularly in terms of how music interacts with different brain regions via its various structural components. Therefore, future research will explore more complex musical structures and design more detailed experimental paradigms, aiming to delve into the relationships between different musical elements and brain functional areas.

(3). While the SSAM model has been shown to be effective, its training phase involves processing a large number of parameters, which slows the learning process. Therefore, the next goal is to reduce the model’s time complexity without sacrificing classification accuracy.

(4). Although the prominent DE feature in EEG recognition was utilized, the optimal feature set specifically for music recall and composition states has not yet been determined. Identifying these features will be a focus of future research, aiming to more precisely recognize and analyze these specific cognitive states.

## Figures and Tables

**Table 1:** Participants and information about the paradigm.

Experiment Information	
Number of participants	24
Number of males	12
Number of females	12
Age of participants	22±2.07
Rating scales	Listen, Creation
Recorded signals	32
Sampling frequency	128

**Table 2:** Information about the musical stimuli used in the paradigm.

Audio Stimuli	
Number of videos	8
Audio content	Piano music
Audio duration	16 s
Creation duration	20 s

**Table 3:** Music recall and continuing database format.

Array Name	Array Shape	Array Contents
listen to music	16×8×32×1920	sub×trial×channel×data
recall and continue music	16×8×32×2560	sub×trial×channel×data
labels	16×16	sub×trial

**Table 4:** Performance comparison on the proposed dataset.

Ref/Author	Year	Model	Accuracy (Normal)		Accuracy (Train)	
			Happy	Sad	Happy	Sad
[32]/Liu	2016	BDAE	50.80%	50.80%	53.60%	51.20%
[33]/Rami Alazrai	2018	2D-CLS	50.80%	51.60%	52.30%	51.60%
[34]/Yin Z	2017	SAEs	53.60%	52.30%	53.60%	51.60%
[35]/Hao Tang	2017	Bimodal-LSTM	57.03%	54.80%	58.59%	57.03%
[36]/Xian LI	2017	Multiple Classifiers Fusion	54.80%	54.80%	57.03%	58.59%
[37]/N.L	2017	ResNet-50 + MFCC	60.16%	58.59%	62.50%	60.16%
[38]/Yilong Yang	2018	3DCNN	77.19%	75.00%	75.00%	75.00%
[39]/Chen	2020	Casc-CL	80.33%	75.00%	80.33%	77.19%
[29]/Wei Tao	2020	ACRNN	83.33%	83.33%	85.00%	83.33%
[22]/Li	2022	SFCSAN	89.75%	85.25%	91.75%	89.84%
Proposed	2023	SSAM	**91.30%**	**89.94%**	**93.14%**	**92.80%**

The highest accuracy in each column is indicated in bold.

**Table 5:** Comparison with different metrics on the proposed dataset.

Model	Accuracy (All)	Precision (All)	Specificity (All)
BDAE	51.60%	51.60%	50.80%
2D-CLS	51.58%	52.30%	50.00%
SAEs	52.78%	51.60%	50.80%
Bimodal-LSTM	55.92%	58.59%	53.60%
Multiple Classifiers Fusion	54.80%	54.80%	52.30%
ResNet-50 + MFCC	60.35%	62.50%	58.59%
3DCNN	75.55%	75.00%	67.03%
Casc-CL	78.21%	75.00%	69.16%
ACRNN	83.75%	77.19%	75.00%
SFCSAN	89.15%	82.91%	80.33%
SSAM	**91.80%**	**89.09%**	**88.37%**

The highest accuracy in each column is indicated in bold.

**Table 6:** Significance analysis of differential entropy features across EEG channels in untrained subjects.

Emotion	Feature	Electrode										
		FP1	FPZ	FP2	FC1	C4	M2	P7	POZ	O1	OZ	O2
Happy	DE-θ	0.337	0.355	0.412	0.735	0.451	0.931	0.271	0.928	0.532	0.454	0.817
	DE-α	0.034	0.015	0.107	0.853	0.151	0.913	0.894	0.252	0.062	0.031	0.956
	DE-β	0.867	0.850	0.759	0.652	0.087	0.862	0.611	0.915	0.817	0.047	0.987
	DE-γ	0.043	0.961	0.629	0.833	0.023	0.744	0.998	0.702	0.551	0.033	0.457
Sad	DE-θ	0.238	0.313	0.861	0.737	0.573	0.487	0.753	0.955	0.861	0.740	0.892
	DE-α	0.011	0.944	0.351	0.613	0.220	0.737	0.364	0.877	0.471	0.021	0.032
	DE-β	0.457	0.361	0.879	0.754	0.216	0.073	0.832	0.879	0.798	0.680	0.729
	DE-γ	0.479	0.832	0.262	0.072	0.615	0.024	0.819	0.867	0.943	0.60.	0.061

Orange shading indicates values with statistical significance (*p* < 0.05), while green shading highlights the key electrodes and features.

**Table 7:** Significance analysis of differential entropy features across EEG channels in trained subjects.

Emotion	Feature	Electrode										
		FP1	FPZ	FP2	FC1	T8	M2	P7	POZ	O1	OZ	O2
Happy	DE-θ	0.073	0.134	0.516	0.871	0.361	0.892	0.539	0.945	0.370	0.761	0.087
	DE-α	0.042	0.073	0.852	0.063	0.955	0.267	0.842	0.796	0.099	0.004	0.727
	DE-β	0.082	0.143	0.585	0.787	0.730	0.101	0.829	0.704	0.712	0.571	0.877
	DE-γ	0.779	0.011	0.107	0.222	0.021	0.073	0.098	0.796	0.047	0.063	0.023
Sad	DE-θ	0.248	0.083	0.852	0.728	0.902	0.339	0.950	0.313	0.859	0.064	0.001
	DE-α	0.165	0.009	0.749	0.170	0.141	0.380	0.106	0.200	0.053	0.131	0.048
	DE-β	0.035	0.033	0.307	0.463	0.478	0.879	0.346	0.105	0.652	0.473	0.192
	DE-γ	0.011	0.857	0.071	0.776	0.015	0.008	0.861	0.520	0.055	0.016	0.007

Orange shading indicates values with statistical significance (*p* < 0.05), while green shading highlights the key electrodes and features.

## Data Availability

The data that support the findings of this study are available upon request from the corresponding author. The data are not publicly available due to privacy.
